# Screening of Oral Squamous Cell Carcinoma Through Color Intensity-Based Textural Features

**DOI:** 10.7759/cureus.56682

**Published:** 2024-03-22

**Authors:** Preethi N Sharma, Minal Chaudhary, Shraddha A Patel, Prajakta R Zade

**Affiliations:** 1 Oral and Maxillofacial Pathology, Sharad Pawar Dental College and Hospital, Datta Meghe Institute of Higher Education and Research, Wardha, IND; 2 Oral Medicine and Radiology, Sharad Pawar Dental College and Hospital, Datta Meghe Institute of Higher Education and Research, Wardha, IND; 3 Dentistry, Indira Gandhi Government Medical College and Hospital, Nagpur, IND

**Keywords:** matlab, homogeneity, energy, entropy, dysplasia, diagnosis, cytology, correlation, contrast, cancer

## Abstract

Background

Early screening and diagnosis of oral squamous cell carcinoma (OSCC) has always been a major challenge for pathologists. Artificial intelligence (AI)-assisted screening tools can serve as an adjunct for the objective interpretation of Papanicolaou (PAP)-stained oral smears.

Aim

This study aimed to develop a handy and sensitive computer-assisted AI tool based on color-intensity textural features to be applied to cytologic images for screening and diagnosis of OSCC.

Methodology

The study included two groups consisting of 80 OSCC subjects and 80 control groups. PAP-stained smears were collected from both groups. The smears were analyzed in Matlab software computed data and color intensity-based textural features such as entropy, contrast, energy, homogeneity, and correlation, were quantitatively extracted.

Results

In this study, a statistically significant difference was noted for entropy, energy, correlation, contrast, and homogeneity. It was found that entropy and contrast were found to be higher with a decrease in homogeneity, correlation, and energy in OSCC when compared to the control group. Receiver operating characteristic curve analysis was done and accuracy, sensitivity, and specificity were found to be 88%, 91%, and 81%, respectively.

Conclusion

The gray-level co-occurrence matrix (GLCM) color intensity-based textural features play a significant role in differentiating dysplastic and normal cells in the diagnosis of OSCC. Computer-aided textural analysis has the potential to aid in the early detection of oral cancer, which can lead to improved clinical outcomes.

## Introduction

Oral cancer accounts for the sixth most common cancer worldwide [1]. It represents approximately 5% of all cancers universally, with 60,000 fresh cases of oral cancer reported annually in India [2]. Oral cancer is linked with alteration in genes that play a significant role in regulating cell growth and apoptosis preceding uncontrolled multiplication of tumor cells, which occur due to exposure to betel quid, tobacco, alcohol, etc. [1]. Alarming changes have been noticed recently in the presentation of oral cancer in younger age groups i.e., those around 40 years or younger with a significant percentage of them having no habits, which is both cryptic and troublesome [1]. The epithelium of the oral mucosa comprises a definite arrangement of cells. When a cell is altered by cancer, the cellular and nuclear morphology gets affected. The oral pathologist usually gives the diagnosis of cancer by observing the biopsy slides of the patient. A biopsy followed by histopathological reporting is an ideal method for diagnosing cancer. Many times it may not be practical to undergo a biopsy in every case as few of the patients may be medically compromised and a few with asymptomatic lesions may not be ready to give their consent for a biopsy [3]. Oral cancers in the early stage are usually asymptomatic, so grievously most of them are symptomatic and advanced at the time of diagnosis [4]. It is difficult to detect them in earlier stages without a thorough head and neck examination by a medical or dental professional. The late diagnosis increases the mortality rate, with less than 50% of patients getting cured. Early detection of oral lesions can increase the survival rate and morbidity thus improving the patient’s quality of life. Cytology is a quite reasonable, simple, painless, and harmless technique well accepted by the patient and a good alternative for the initial diagnosis of oral cancer [5]. The methodology involves the Papanicolaou (PAP) test which demands microscopic analysis of exfoliated cells from the oral epithelium. The ease of using a toothbrush in the oral cavity yields cells from deep layers of the epithelial surface [6,7]. Reviewing the PAP smear slides has become more time-consuming and skillful judgment is required for the same. It also increases the workload of the pathologists leading to their fatigue. In addition, the report may also be observer-biased many times. In light of these challenges, an image processing approach i.e., to develop computer-assisted software that will diagnose the initial stages of oral cancer is the call of the hour, especially in a developing country like India. It will be of huge assistance in laboratories with high patient volume burdens and in the cancer screening camps where the maximum number of cases may be normal and the pathologist may focus more on the cases diagnosed as malignant by the system. Hence, the development of computer-assisted software, which screens the abnormal cells automatically, is both relevant and significant.

Generally, automated screening is processed by obtaining a specific set of pertinent features from the obtained images. Usually, a data set is formed and trained. In general, an image entails shape, color, and texture features. In medical computing imaging, gray-level images are quite popular [8]. The shape and size-based features are helpful but analyzing them depends on the precise segmentation of the cells and their nuclei. In the literature of medical image processing, numerous methods of segmentation methods have been studied but they witness several problems. The accuracy of these algorithms relies on multiple parameters and may not be appropriate for all types of images. Even a minor inefficiency in segmentation may mispresent the shape feature prompting less accuracy [9]. However textural features on the other hand can be obtained precisely. They are highly applicable to the microscopic structures in the cell that are disclosing signs of cancer. Studies on texture analysis of oral PAP smear images in the Indian population are very limited in the literature. There is a direct need to detect oral squamous cell carcinoma (OSCC) through artificial intelligence (AI) for early diagnosis and reduction in the mortality rate. The present study is an attempt to develop a handy and sensitive computer-assisted AI tool based on gray-level co-occurrence matrix (GLCM) color intensity textural features to be applied to cytologic images for screening and diagnosis of OSCC.

## Materials and methods

This study was conducted in the School of Oral Oncology, Department of Oral Pathology and Microbiology, Sharad Pawar Dental College and Hospital, Datta Meghe Institute of Higher Education and Research, Wardha, India along with assistance from Datta Meghe Institute of Engineering, Technology and Research, Wardha, India. This study was a prospective audit for which the necessary Institutional Ethics Committee, Datta Meghe Institute of Medical Sciences approval (Ref. No. DMIMS(DU)/IEC/2017-18/6461) was obtained. The study population was obtained from the OPD of the Department of Oral Pathology and Microbiology.

Inclusion criteria

The study included subjects who have given written informed consent, a control group without tobacco habit, and subjects clinically diagnosed with OSCC (T1N0M0).

Exclusion criteria

The study excluded subjects who had undergone treatment for malignancy, subjects with recurrence of OSCC, subjects with co-existing malignancy, immune-compromised subjects, and those suffering from chronic debilitating diseases such as diabetes and tuberculosis.

Methodology

The study included two groups consisting of 80 OSCC subjects and 80 control groups. Oral exfoliative cytology was performed using a nylon toothbrush in all the selected patients. The entire procedure was explained to the patients and informed consent was obtained from the patients. The patients were asked to swish their oral cavity with water to clean off debris and the area of suspicion was cleaned with a swab and then a smear was taken. A total of three smears were taken from each patient and smears with a good density of cells were taken for analysis. The toothbrush was stroked in one direction repeatedly over the area of the lesion till the appearance of pinpoint reddish areas, thus attaining the epithelial cells through the entire epithelial thickness. The smear was spread on the dried glass slide, a fixative was sprayed, and staining was performed by the modified rapid PAP staining method (BioLab Diagnostics, Mumbai, India). The stained smears were observed under ×40 magnification using a camera-fitted LEICA DMLB2 research microscope. Overlapped, clumped, and folded cells were not taken into consideration for the analysis. We looked for suitable density of cells showing dysplastic features such as adequacy of abnormal cells, nuclear and cellular pleomorphism, hyperchromatism, and nuclear-cytoplasmic ratio. A total of 160 smears were considered for analysis in the study. About 10 manually cropped images were taken from each smear. A total of 1600 cropped images were considered in the study. The results of a double-blind examination of each smear performed independently by two different cytopathologists were recorded. The results were classified considering the scoring system based on dysplastic changes as 0 representing "normal with no epithelial abnormality" and 1 representing "epithelial dysplasia." Slides with significant hemorrhage, paucity of cells, or inadequate staining were disregarded.

**Figure 1:**
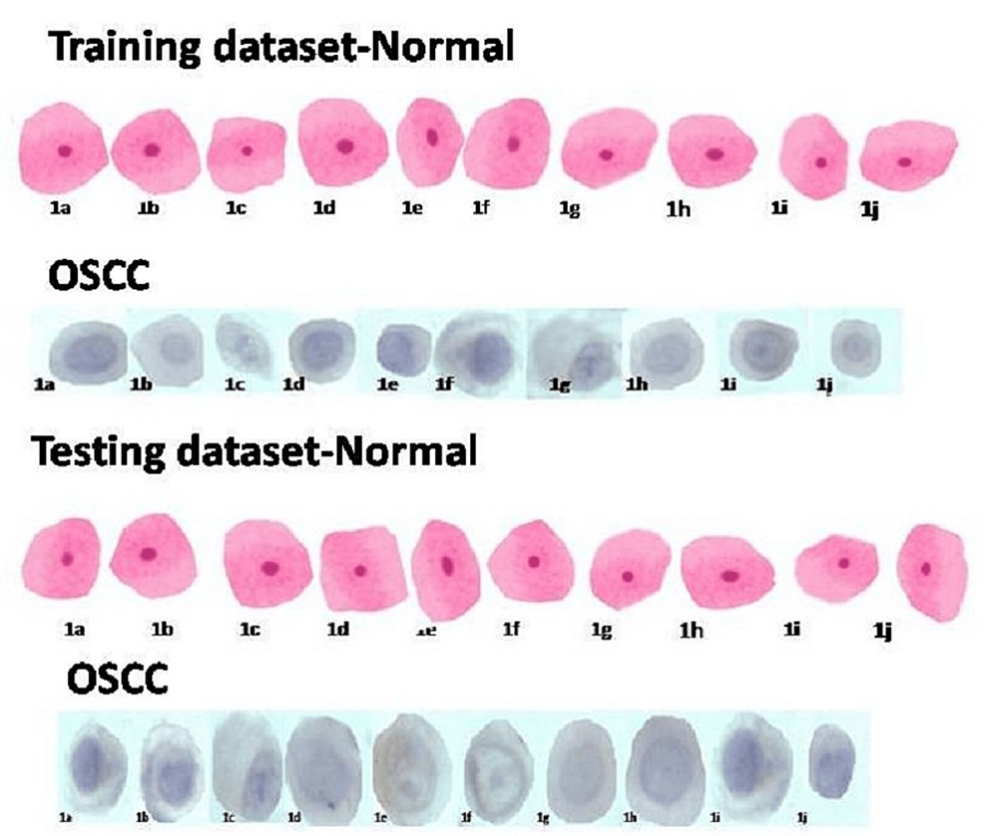
Training dataset and testing dataset OSCC: Oral squamous cell carcinoma

A database comprising PAP smear cell images of normal and dysplastic cells appropriately scored was collected from the cytology archives of the Department of Oral Pathology and was trained in the Matlab high-level programming software before processing the study data as there was no benchmark database available. A total of 100 smears out of which 50 normal and 50 OSCC smears were taken from the archives of the department. A total of 10 cells were cropped from each smear and trained. The pathologist has marked the “region of interest (ROI)” from each slide. The training involved passing the training dataset (labeled) to the model and adjusting its parameters until it reaches a level of accuracy that is satisfactory (the training dataset was carefully curated) (Figure 1).

The following steps were involved in Matlab Processing Software 1. Image preprocessing - contrast enhancement of the ROI of the captured image was the main purpose of the preprocessing. 2. Image segmentation - an image is segmented into its areas or objects. In this study, the images were manually cropped after selecting the ROI and a cytology dataset was prepared (Figure 1). The desired information was preserved using the fuzzy c-means (FCM) segmentation approach. FCM segmentation provides better performance than other clustering algorithms when dealing with complex data distributions and hence this segmentation was used in this study. After segmentation, the segmented image contained the actual information that was utilized to quantitatively extract all the feature information. 3. Feature extraction - it is one of the most important steps in the analysis of cytology pap smear images. Cellular-level microscopic images were used for this step. It determines various attributes as well as properties associated with a region or object. Feature extraction isolates distinct portions and features of images. Here in this study, GLCM-based color intensity textural features such as entropy, energy, contrast, correlation, and homogeneity were extracted from the given input images. Textural features are computationally efficient and scalable compared to morphological features and hence textural features were considered in the study. The images were analyzed in MATLAB software computed data with some useful algorithms followed by validation. The results were presented with a detailed analysis of textural features in individual groups as descriptive statistics and their significant differences in the groups.

Statistical analysis

The software used for statistical analysis was IBM SPSS Statistics for Windows, Version 24 (Released 2016; IBM Corp., Armonk, New York, United States) and GraphPad Prism 7.0 version, and p<0.05 is considered as a level of significance. Data was presented by mean and standard deviation. Descriptive statistics using students' unpaired t-tests were applied between the groups.

## Results

**Figure 2:**
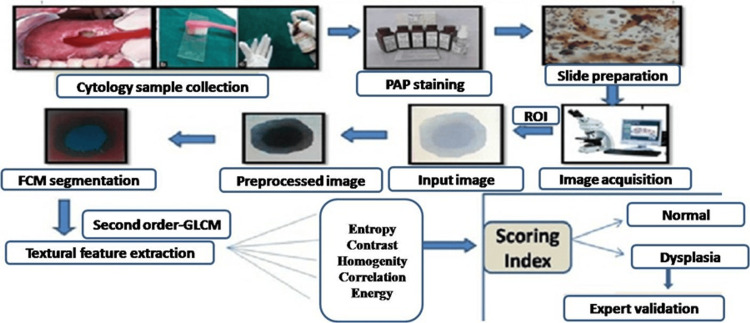
Working of the study PAP: Papanicolaou; FCM: Fuzzy c-means; ROI: Region of interest; GLCM: Grey level co-occurrence matrix

Figure 2 depicts the working of the study comprising of cytology sample collection, PAP staining, slide preparation, image acquisition, textural feature extraction, scoring index, and validation.

Table 1 depicts the comparison of textural features such as entropy, contrast, energy, correlation, and homogeneity with mean values and significant p-values. Entropy and contrast were found to be increased with a decrease in energy, correlation, and homogeneity in OSCC when compared to the control.

**Table 1:** Comparison of textural features such as entropy, contrast, energy, correlation, and homogeneity in OSCC and control OSCC: Oral squamous cell carcinoma

Textural features	OSCC (N=80)		Control (N=80)		t-value p-value	
	Mean	Standard deviation	Mean	Standard deviation		
Entropy	2.05	0.19	1.84	0.04	9.68	0.0001,S
Contrast	0.11	0.03	0.06	0.01	11.17	0.0001,S
Energy	0.38	0.04	0.40	0.02	2.68	0.006,S
Correlation	0.94	0.015	0.95	0.006	4.36	0.0001,S
Homogeneity	0.94	0.005	0.95	0.014	3.82	0.0001,S

**Figure 3:**
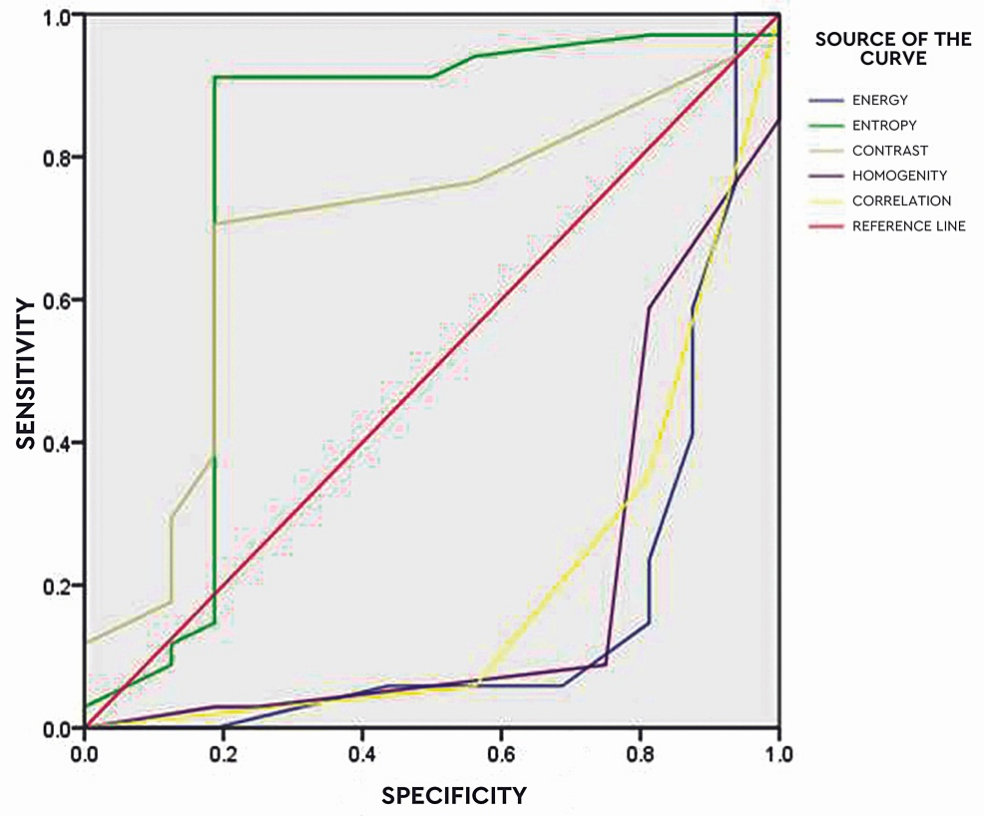
Receiver operating characteristic curve analysis

Figure 3 depicts the receiver operating characteristic (ROC) curve analysis. The parameters like accuracy, sensitivity, and specificity of all features were found to be 88%, 91%, and 81%, respectively.

## Discussion

OSCC is the bane of the Indian subcontinent. OSCC involves the epithelium of the oral cavity [10]. The early cancer detection presents a significant challenge for pathologists and general practitioners, impacting diagnosis, treatment planning, and subsequent care across all stages of the disease [11]. Numerous methods are used for diagnosis and screening, cytology is one of them. Obtaining and analyzing smears from the huge target population addicted to tobacco is a task of the mammoth population. Screening and analysis require trained professionals, skilled enough to identify a handful of dysplastic cells among a few hundred thousand cells. New automated technologies are being developed for the same but are found to be expensive as they are trademarked and not endemically developed software catering to our population's needs. Numerous means of automation have been applied; however, image analysis appears to be the most promising of them. The development of an image-processing approach helps identify altered cells which are the hallmarks of oral malignancy. This addresses a pressing priority, particularly in an advancing country like India where the doctor and patient ratio especially in remote areas is minimal. It will also be of huge assistance in labs with high volume workloads and in cancer screening programs to optimize the workload on pathologists.

The research on the application of AI has been profound in the cytology of cervical cancer [12]. However, there are very few studies regarding AI applications in OSCC [11]. Most analytical studies to date have been carried out using clinical, radiographic, and histopathological images. There is a paucity of analysis based on digitized parameters. Thus, there is a necessity to develop an AI-aided tool for preliminary diagnosis of OSCC by way of screening oral cytology PAP smears. Exfoliative cytology is a simple, painless, and convenient technique accepted by the patient and perfect for the initial screening and diagnosis of oral cancer especially since it is not practical to perform the biopsy in every case. Also, patients who are medically compromised or with asymptomatic lesions may not provide their consent for biopsy but often agree to cytology as it is non-invasive. Taking these factors into consideration was identified a need for an AI-aided screening method for OSCC.

Many GLCM elements have very small values when the image's texture is not uniform, which suggests that the entropy is quite high [13]. The mean entropy value in our study was found to be higher in cells with OSCC when compared to the control group. Contrast is the amount of local variations present in an image. If each pair of pixels has a different grey level, the contrast is anticipated to be high. The mean contrast value in our study was found to be higher in cells with OSCC when compared to the control group. The findings are similar to a study conducted by Lian et al. in 2018 where an optical method with a Scanned Laser Pico-projection system (SLPP) and GLCM on tissue sections of OSCC was used [14]. Several factors contribute to the increase in entropy and contrast in cancer cells [15,16]. One of the main reasons is the abnormal metabolism that occurs in cancer cells. Another factor that contributes to the increase in entropy and contrast is the genetic mutations and alterations that occur in these cells.

When an image has excellent homogeneity or when the pixels are substantially comparable, energy is high. The mean energy value in our study was found to be lower in cells with OSCC when compared to the control group. The linear relationship between the grey levels of adjacent pixels is measured through correlation. The mean correlation value in our study was found to be lower in cells with OSCC when compared to the control group. Homogeneity measures the local homogeneity of a pixel pair. The mean homogeneity value in our study was found to be lower in cells with OSCC when compared to the control group. These findings are similar to a study conducted by Lian et al. in 2018 where an optical method with a SLPP and GLCM on tissue sections of OSCC was used [14]. The present study found a decrease in energy, homogeneity, and correlation in OSCC when compared to the control group. Overall, the decrease in energy, correlation, and homogeneity in OSCC is the result of improper functioning and dysregulation of the various cellular processes [15,16].

The idea of formulation of the scoring index was taken from the article based on the oxidative stress index and the reference article is included [17]. A score ranging below 1 indicated a normal cell and a score above 1 indicated a malignant cell. For validation of the AI-aided application, the accepted reference standards for cytological evaluation were used by two different cytopathologists to independently review each smear in a double-blind method. The results were recorded considering the grading system as 0 representing "normal" and 1 representing "epithelial dysplasia." Based on the analysis conducted, no difference was recorded in diagnosis between the two observers. The recorded output was then compared with the output of the computerized software and the entropy, contrast, energy, correlation, and homogeneity values were noted. ROC analysis was applied to assess the overall diagnostic performance of the software. The parameters like accuracy, sensitivity, and specificity of all features were found to be 88%, 91%, and 81%, respectively.

## Conclusions

The GLCM color intensity-based textural features play a significant role in differentiating dysplastic and normal cells in the diagnosis of OSCC. In this study increased values of entropy and contrast along with decreased energy, correlation, and homogeneity were found in PAP-stained cells of OSCC when compared to normal. The algorithm developed in this software can be incorporated with the microscope equipment so that the pathologist can view the slides and record the numerical values on manually cropped images. There were certain technological limitations in the study. Computer-aided textural analysis has the potential to aid in the early detection of oral cancer, which can lead to improved clinical outcomes. Further research is needed to expand the techniques and algorithms used in this analysis and to conduct large-scale clinical trials to validate their effectiveness in clinical practice.

## References

[1] Warnakulasuriya S (2009). Global epidemiology of oral and oropharyngeal cancer. Oral Oncol.

[2] Ghosh G, Jayaram KM, Patil RV, Malik S (2011). Alterations in serum lipid profile patterns in oral squamous cell carcinoma patients. J Contemp Dent Pract.

[3] Babshet M, Nandimath K, Pervatikar S, Naikmasur V (2011). Efficacy of oral brush cytology in the evaluation of the oral premalignant and malignant lesions. J Cytol.

[4] Mehrotra R, Gupta A, Singh M, Ibrahim R (2006). Application of cytology and molecular biology in diagnosing premalignant or malignant oral lesions. Mol Cancer.

[5] Divani S, Exarhou M, Leonidas-Nectarios T, Georgantzis D, Skoulakis H (2009). Advantages and difficulties of brush cytology in the identification of early oral cancer. Arch Oncol.

[6] Rick GM (2003). Oral brush biopsy: the problem of false positives. Oral Surg Oral Med Oral Pathol Oral Radiol Endod.

[7] Svirsky JA, Burns JC, Page DG, Abbey LM (2001). Computer-assisted analysis of the oral brush biopsy. Compend Contin Educ Dent.

[8] Myler HR, Weeks AR (1993). Computer Imaging Recipes in C. Prentice-Hall, Inc.USA.

[9] Jayasingh E, Allwin S (2013). Detection of cancer in pap smear cytological images using bag of texture features. IOSR J Comput Eng.

[10] Bugshan A, Farooq I (2020). Oral squamous cell carcinoma: metastasis, potentially associated malignant disorders, etiology and recent advancements in diagnosis. F1000Res.

[11] González-Moles MÁ, Aguilar-Ruiz M, Ramos-García P (2022). Challenges in the early diagnosis of oral cancer, evidence gaps and strategies for improvement: a scoping review of systematic reviews. Cancers (Basel).

[12] Hou X, Shen G, Zhou L, Li Y, Wang T, Ma X (2022). Artificial intelligence in cervical cancer screening and diagnosis. Front Oncol.

[13] Zubair AR, Alo OA (2019). Grey level co-occurrence matrix (GLCM) based second order statistics for image texture analysis. Int J Sci Eng Investig.

[14] Lian MJ, Huang CL, Lee TM (2018). Automation characterization for oral cancer by pathological image processing with gray-level co-occurrence matrix. J Image Graph.

[15] Wohl I, Sherman E (2019). ATP-dependent diffusion entropy and homogeneity in living cells. Entropy (Basel).

[16] Vander Heiden MG, Cantley LC, Thompson CB (2009). Understanding the Warburg effect: the metabolic requirements of cell proliferation. Science.

[17] Sánchez-Rodríguez MA, Mendoza-Núñez VM (2019). Oxidative stress indexes for diagnosis of health or disease in humans. Oxid Med Cell Longev.

